# Low Baseline Pulmonary Levels of Cytotoxic Lymphocytes as a Predisposing Risk Factor for Severe COVID-19

**DOI:** 10.1128/mSystems.00741-20

**Published:** 2020-09-01

**Authors:** Pascal H. G. Duijf

**Affiliations:** a Institute of Health and Biomedical Innovation, Queensland University of Technology (QUT), Faculty of Health, School of Biomedical Sciences, Brisbane, QLD, Australia; b Centre for Data Science, Queensland University of Technology (QUT), Brisbane, QLD, Australia; c University of Queensland Diamantina Institute, The University of Queensland, Translational Research Institute, Brisbane, QLD, Australia; Oxford Nanopore Technologies

**Keywords:** COVID-19, SARS-CoV-2, ACE2, TMPRSS2, T cells, NK cells

## Abstract

Coronavirus disease 2019 (COVID-19) is caused by the severe acute respiratory syndrome coronavirus 2 (SARS-CoV-2) and currently has detrimental human health, community, and economic impacts around the world. It is unclear why some SARS-CoV-2-positive individuals remain asymptomatic, while others develop severe symptoms. Baseline pulmonary levels of antiviral leukocytes, already residing in the lung prior to infection, may orchestrate an effective early immune response and prevent severe symptoms. Here, “*in silico* flow cytometry” was used to deconvolute the levels of all seven types of antiviral leukocytes in 1,927 human lung tissues. Baseline levels of CD8^+^ T cells, resting NK cells, and activated NK cells, as well as cytokines that recruit these cells, are significantly lower in lung tissues with high expression of the SARS-CoV-2 entry receptor angiotensin-converting enzyme 2 (ACE2). This is observed in univariate analyses, in multivariate analyses, and in two independent data sets. Importantly, ACE2 mRNA and protein levels very strongly correlate in human cells and tissues. The above findings also largely apply to the SARS-CoV-2 entry protease TMPRSS2. Both SARS-CoV-2-infected lung cells and COVID-19 lung tissues show upregulation of CD8^+^ T cell- and NK cell-recruiting cytokines. Moreover, tissue-resident CD8^+^ T cells and inflammatory NK cells are significantly more abundant in bronchoalveolar lavage fluids from mildly affected COVID-19 patients compared to severe cases. This suggests that these lymphocytes are important for preventing severe symptoms. Elevated ACE2 expression increases sensitivity to coronavirus infection. Thus, the results suggest that some individuals may be exceedingly susceptible to develop severe COVID-19 due to concomitant high preexisting ACE2 and TMPRSS expression and low baseline cytotoxic lymphocyte levels in the lung.

**IMPORTANCE** COVID-19 is caused by the highly contagious coronavirus SARS-CoV-2 and currently has detrimental human health, community, and economic impacts around the world. It is unclear why some SARS-CoV-2-positive individuals develop severe COVID-19 symptoms, which can be fatal, while others only develop mild symptoms. In the absence of an effective and widely available vaccine, it is of paramount importance that we identify risk factors for development of severe symptoms to be able to improve treatment approaches. The *ACE2* gene encodes the receptor on human cells that the virus uses to infect these cells. This study finds that if the lungs of healthy individuals have high levels of ACE2, they typically have low levels of the immune cells that eliminate viruses. Therefore, some individuals may develop severe COVID-19 due to simultaneous high levels of the virus receptor and low levels of immune cells that eradicate the virus in their lungs.

## Abstract (precis)

COVID-19 is caused by the highly contagious coronavirus SARS-CoV-2 and currently has detrimental human health, community, and economic impacts around the world. It is unclear why some SARS-CoV-2-positive individuals develop severe COVID-19 symptoms, which can be fatal, while others only develop mild symptoms. In the absence of an effective and widely available vaccine, it is of paramount importance that we identify risk factors for development of severe symptoms to be able to improve treatment approaches. The *ACE2* gene encodes the receptor on human cells that the virus uses to infect these cells. This study finds that if the lungs of healthy individuals have high levels of ACE2, they typically have low levels of the immune cells that eliminate viruses. Therefore, some individuals may develop severe COVID-19 due to simultaneous high levels of the virus receptor and low levels of immune cells that eradicate the virus in their lungs.

## INTRODUCTION

Coronaviruses are viruses belonging to the family *Coronaviridae* (1). They are large, single-stranded RNA viruses that often originate from bats and commonly infect mammals. While the majority of coronavirus infections cause mild symptoms, some can cause severe symptoms, such as pneumonia, respiratory failure and sepsis, which may lead to death (2, 3).

Coronavirus zoonosis constitutes a serious health risk for humans. Indeed, in recent history, transmissions of three types of coronaviruses to humans have led to various numbers of deaths. The outbreak of the severe acute respiratory syndrome (SARS) epidemic, which is caused by the SARS coronavirus (SARS-CoV), originated in Guangdong, China in 2002 and led to nearly 800 deaths (4). The Middle East respiratory syndrome coronavirus (MERS-CoV) outbreak, which emerged in Saudi Arabia in 2012, similarly caused about 800 deaths but with more than 8,000 cases, nearly four times as many cases were reported (4). Finally, coronavirus disease 19 (COVID-19), caused by severe acute respiratory syndrome coronavirus 2 (SARS-CoV-2), is currently causing a pandemic. On 1 May 2020, the World Health Organization reported over 3 million confirmed cases and over 220,000 patients who have succumbed to COVID-19 around the world (5). However, the actual number of deaths is probably considerably higher (6). In addition, this figure is still soaring; on 1 May 2020, the rate exceeded 6,400 deaths per day (5).

To infect target cells, coronaviruses use their spike (S) glycoprotein to bind to receptor molecules on the host cell membrane. Angiotensin-converting enzyme 2 (ACE2) has been identified as the main SARS-CoV-2 entry receptor on human cells (7, 8), while the serine protease TMPRSS2, or potentially cathepsin B and L, are used for S-protein priming to facilitate host cell entry (7). SARS-CoV-2 S protein has a 10- to 20-fold-higher affinity to human ACE2 than SARS-CoV S protein (9). Moreover, ACE2 expression proportionally increases the susceptibility to S protein-mediated coronavirus infection (10–12). Hence, increased expression of ACE2 is thought to increase susceptibility to COVID-19 (13–15).

Epithelial cells of the respiratory tract, including the lung, are primary SARS-CoV-2 target cells (16–18). These cells can sense viral infection via pattern recognition receptors (PRRs). PRRs, including Toll-like receptors and NOD-like receptors, recognize pathogen-associated molecular patterns (PAMPs) (19). Upon PRR activation, a range of proinflammatory cytokines and chemokines are produced and released in order to activate the host’s immune system. Interferons (IFNs), in particular type I and type III IFN, are among the principal cytokines to recruit immune cells (19, 20).

Six types of leukocytes have been implicated in detecting and responding to viral infections in the lung, a major site of SARS-CoV-2 infection, which also presents with severe COVID-19 symptoms. The cytotoxic activities of CD8^+^ T cells and NK cells can facilitate early control of viral infections by clearing infected cells and avoiding additional viral dissemination (21, 22). Dendritic cells specialize in sensing infections, including by viruses, and inducing an immune response (23). CD4^+^ T cells contribute to viral clearance by promoting production of cytokines and interactions between CD8^+^ T cells and dendritic cells (24). M1 macrophages interact with pulmonary epithelial cells to fight viral infections in the lung (25). Finally, neutrophils may contribute to clearance of viral infections through phagocytosis of virions and viral particles. However, their precise role is uncertain (26).

SARS-CoV-2 is considerably more efficient in infection, replication, and production of infectious virus particles in human lung tissue than SARS-CoV (17). Strikingly, despite this, SARS-CoV-2 initially does not significantly induce type I, II, or III IFNs in infected human lung cells and tissue (17, 27). When this does occur, it may in fact promote further SARS-CoV-2 infection, as IFNs directly upregulate expression of the SARS-CoV-2 receptor ACE2 (28). These observations suggest that baseline levels of leukocytes, which already reside in the lung prior to infection, may be important in mounting a rapid immune response against SARS-CoV-2 infection and prevent severe COVID-19 symptoms. As stated above, ACE2 expression level may be a predictor of increased susceptibility to COVID-19 (10–15). Thus, here I investigated the relationship between ACE2 and TMPRSS2 expression and the levels of seven leukocyte types implicated in antiviral immune response in human lung tissue.

## RESULTS

**FIG 1:**
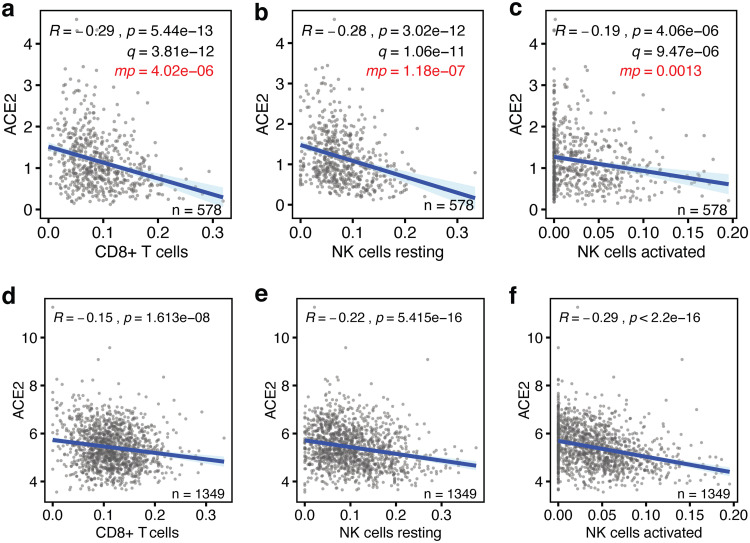
Baseline levels of cytotoxic lymphocytes inversely correlate with ACE2 expression in the lung. (a to c) The correlations between the baseline levels of CD8^+^ T cells, resting NK cells, and activated NK cells (*x* axes) and those of the SARS-CoV-2 host cell receptor ACE2 (*y* axes) in human lung tissue are shown. Data are from the GTEx data set (*n* = 578). (d to f) The same correlations are shown for lung tissues in the LUG data set (*n* = 1,349). Regression lines and 95% confidence intervals are shown. Pearson correlation *R* and *P* values are shown, *q* values are Benjamini-Hochberg-adjusted *P* values using a false discovery rate of 0.05, and *mp* values are multivariate *P* values (Table 1).

I used bulk transcriptome sequencing (RNAseq) gene expression data from the 578 human lung tissues present in the Genotype-Tissue Expression (GTEx) database (29, 30), because this is the largest publicly available data set with clinical information. Using an established “*in silico* flow cytometry” pipeline (31), I estimated the levels of CD8^+^ T cells, resting and activated NK cells, M1 macrophages, dendritic cells, CD4^+^ T cells, and neutrophils in these tissues (see Fig. S1a to c and Table S1 in the supplemental material). These levels were compared to ACE2 expression levels in these lung tissues. This revealed that ACE2 expression is negatively correlated with the levels of CD8^+^ T cells, resting and activated NK cells, and M1 macrophages (*P* < 8 × 10^−6^, Pearson correlations) (Fig. 1a to c). However, there are no statistically significant correlations between ACE2 expression and the levels of CD4^+^ T cells, dendritic cells, and neutrophils (*P* > 0.05) (Fig. S2a to d). Thus, the levels of a majority of leukocytes involved in antiviral immune responses are significantly lower in lung tissues with high ACE2 expression levels.

10.1128/mSystems.00741-20.2 FIG S1 *In silico* cytometry statistics. Per-sample statistics of *in silico* cytometry on human lung tissues are shown for the GTEx (*n* = 578) and LUG (*n* = 1,349) data sets. (a) Pearson correlation coefficients *R*. (b) Root mean square errors (RMSE). (c) *P* values. *P* values < 2.2 × 10^−16^ were processed as equal to 2.2 × 10^−16^. Source data are provided in Table S1. Download FIG S1, PDF file, 0.6 MB. Copyright © 2020 Duijf. 2020 Duijf This content is distributed under the terms of the Creative Commons Attribution 4.0 International license.

10.1128/mSystems.00741-20.7 TABLE S1 Source data of *in silico* cytometry statistics. Download Table S1, DOCX file, 0.2 MB. Copyright © 2020 Duijf. 2020 Duijf This content is distributed under the terms of the Creative Commons Attribution 4.0 International license.

10.1128/mSystems.00741-20.3 FIG S2 Correlations between baseline antiviral leukocyte levels and ACE2 expression levels in human lung tissues. (a to d) The correlations between the baseline levels of indicated immune cell types (*x* axes) and the expression level of the SARS-CoV-2 host cell receptor ACE2 (*y* axis) in human lung tissue are shown. Data are from the GTEx data set (*n* = 578). Regression lines and 95% confidence intervals are shown. *R* and *P* values are Pearson correlations; *q* values are Benjamini-Hochberg-adjusted *P* values using a false discovery rate of 0.05. See also Fig. 1. Download FIG S2, PDF file, 1.8 MB. Copyright © 2020 Duijf. 2020 Duijf This content is distributed under the terms of the Creative Commons Attribution 4.0 International license.

It is possible that some of the above observations are linked to phenotypic characteristics, such as sex, age, body mass index (BMI), race, or smoking status. To test the robustness of our findings, I applied multivariable regression analysis that includes these five covariates (Table S2), as well as the levels of the above seven leukocyte types or states. This showed that only 4 of the 12 variables significantly contribute to predicting ACE2 expression levels, specifically the levels of CD8^+^ T cells, resting NK cells, activated NK cells, and M1 macrophages (Fig. 1a to c and Table 1). Notably, none of the five added phenotypic covariates showed statistically significant contributions. Consistently, I found limited statistically significant correlations between these variables and ACE2 expression in univariate analyses, irrespective of whether they were analyzed as continuous data or binned into discrete ordinal categories (Fig. S3a to j). Thus, the levels of four types of leukocytes that respond to viral infection are low in lung tissue with high ACE2 expression levels independently of phenotypic covariates.

**TABLE 1:** Multivariate analysis of ACE2 expression in human lung tissue

Variable	*β* value	SE^*a*^	*t* value	*P* value	*P* value symbol^*b*^
CD8^+^ T cells	−2.719	0.584	−4.656	4.02e−06	****
NK cells, resting	−3.128	0.583	−5.366	1.18e−07	****
NK cells, activated	−2.281	0.704	−3.241	0.0013	**
M1 macrophages	−4.963	1.339	−3.707	0.0002	***
CD4^+^ T cells	0.453	0.513	0.882	0.3781	
Dendritic cells	−12.793	6.688	−1.913	0.0563	
Neutrophils	−0.398	0.469	−0.848	0.3971	
Sex = male (vs female)	0.065	0.056	1.16	0.2466	
Age	0.004	0.002	1.799	0.0726	
BMI	−0.004	0.006	−0.556	0.5787	
Race = Caucasian (vs non-Caucasian)	−0.096	0.073	−1.313	0.1899	
Smoking status = yes (vs no)	−0.025	0.058	−0.433	0.6650	

a SE, standard error.

b *P* value symbols: **, *P* < 0.01; ***, *P* < 0.001; ****, *P* < 0.0001.

10.1128/mSystems.00741-20.4 FIG S3 Univariate analyses of phenotypic covariates included in multivariate analyses. Univariate analyses of five covariates that were included in multivariate analyses of ACE2 and TMPRSS2 expression in human lung tissue using the GTEx data set (Table 1 and Table S2). (a) Sex; (b) age; (c) race; (d and e) body mass index; (f to j) Smoking behavior, referring to smoking status (smoker/non-smoker) (f), number of units smoked during the smoke period (g and h), and number of years of smoking (i and j). *P* values in categorical analyses were from Mann-Whitney *U* tests. *R* and *P* values in continuous analyses are Pearson correlations. *P* values in panels h and j are only shown if *P* < 0.05. Sample numbers (*n*) are shown on the *x* axes. Download FIG S3, PDF file, 2.5 MB. Copyright © 2020 Duijf. 2020 Duijf This content is distributed under the terms of the Creative Commons Attribution 4.0 International license.

10.1128/mSystems.00741-20.8 TABLE S2 Phenotypic characteristics of GTEx lung tissue donors. Download Table S2, DOCX file, 0.02 MB. Copyright © 2020 Duijf. 2020 Duijf This content is distributed under the terms of the Creative Commons Attribution 4.0 International license.

Next, whether the above observations could be validated in an independent cohort of individuals was tested. For this, I used, to my knowledge, the largest publicly available lung tissue data set. The Laval University, University of British-Columbia, Groningen University (LUG) data set, including microarray gene expression data of 1,349 human lung tissues, was used. Following determination of ACE2 expression levels and estimation of the levels of CD8^+^ T cells, resting NK cells, activated NK cells, and M1 macrophages (Fig. S1a to c and Table S1), I found that three of the four also negatively correlated with ACE2 expression in this independent data set (*P* < 2 × 10^−8^) (Fig. 1d to f). With a correlation coefficient of *R *= 0.096, only M1 macrophages did not correlate with ACE2 expression in this data set (Fig. S4). Thus, our observations indicate that the baseline levels of three types of cytotoxic lymphocytes, specifically CD8^+^ T cells, resting NK cells, and activated NK cells, are robustly and consistently low in lung tissue with high expression of the SARS-CoV-2 receptor ACE2.

10.1128/mSystems.00741-20.5 FIG S4 Correlation between baseline M1 macrophage levels and ACE2 expression level in human lung tissue. The correlation between these variables is shown as described in Fig. S2. Data are from the LUG data set (*n* = 1,349). Download FIG S4, PDF file, 1.3 MB. Copyright © 2020 Duijf. 2020 Duijf This content is distributed under the terms of the Creative Commons Attribution 4.0 International license.

**FIG 2:**
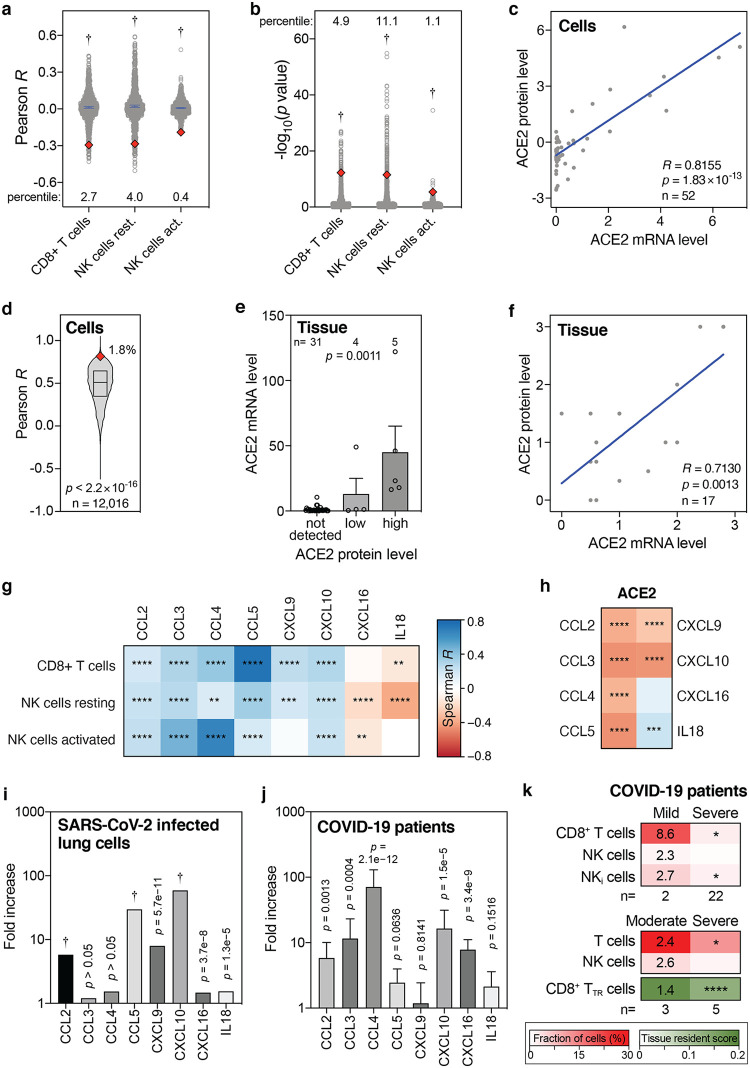
Levels of ACE2 mRNA, ACE2 protein, CD8^+^ T cells, NK cells, and cytokines in lung cells, lung tissues, and COVID-19 patient samples. (a and b) Pearson *R* and −log_10_ *P* values of correlations between 1,000 randomly sampled genes and the levels of indicated lymphocytes in lung tissues were determined and plotted. The NK cells were resting and activated NK cells. ACE2 Pearson *R* and *P* values are shown as red diamonds. Blue lines indicate means with 95% confidence intervals. Percentiles for ACE2 with respect to the 1,000 random *R* and *P* values are shown. Data are from the GTEx data set (*n* = 578). The *P* values are from one-sample *t* tests. (c) Correlations between ACE2 mRNA and protein levels in 52 cell lines. *R* and *P* values are from Pearson correlations. (d) Pearson correlation *R* values between mRNA and protein levels of 12,016 genes are compared to the ACE2 *R* coefficient (red diamond). The line and box represent the median and interquartile ranges. The ACE2 *R* percentile is also shown. The *P* value is from one-sample *t* test. (e) Bar graph showing the correlation between ACE2 mRNA and protein levels in human tissues. Means plus standard errors of the means (error bars) are shown. Samples are from the Human Protein Atlas. *P* values were from Kruskal-Wallis test. (f) Meta-analysis scatterplot showing the correlation between ACE2 mRNA and protein levels in 17 human tissues. Data are from nine different studies, as detailed in Table S3 in the supplemental material. *R* and *P* values are from Pearson correlation. (g and h) Heatmaps showing Spearman correlations between the levels of ACE2 or indicated cytotoxic lymphocytes and eight cytokines that recruit these cells in human lung tissues from the GTEx data set (*n* = 578). The colors of the tiles represent Spearman *R*, per the color bar on the right. Spearman significance levels are shown by asterisks. See also Fig. S5. (i and j) Fold increase in expression levels of indicated cytokines in Calu-3 lung cells 24 h after SARS-CoV-2 infection compared to uninfected Calu-3 cells (i), and in postmortem COVID-19 lung tissues (*n* = 2) to those in healthy, uninfected lung tissues (*n* = 2) (j). (k) Comparison of indicated fractions of lymphocyte levels in bronchoalveolar lavage fluids from mild/moderate and severe COVID-19 patients, as determined in two separate studies (46, 47). The second study also determined a tissue resident (TR) score for CD8^+^ T cells. Numbers in the mild/moderate column on the left show fold increase compared to the respective severe cases on the right. Asterisks in the severe column on the right represent statistical significance levels, as determined by Mann-Whitney *U* tests (top panel), or *t* tests (middle and bottom panels) comparing mild/moderate to severe cases. *P* value symbols: *, *P* < 0.05; **, *P* < 0.01; ***, *P* < 0.001; ****, *P* < 0.0001; †, *P* < 2.2 × 10^−16^.

To more rigorously assess our observations, a range of additional analyses was employed. Although highly statistically significant (all *P* values < 4.1 × 10^−6^, Fig. 1), the absolute Pearson *R* values between baseline levels of ACE2 and the three lymphocyte types seemed to be low, as they ranged between 0.2 and 0.3 (Fig. 1a to c). To test how strong these are in relative terms, the Pearson *R* and *P* values of 1,000 randomly sampled other genes were calculated. This revealed that the ACE2 *R* and *P* values were significantly lower than expected by chance (all one-sample *t* test *P* < 2.2 × 10^−16^; Fig. 2a and b). In addition, these *R* and *P* values ranked in the top 0.4 to 11 percentiles of strongest and most significant correlations for each of the three leukocyte types (Fig. 2a and b). Thus, the seemingly low correlations between ACE2 mRNA and cytotoxic lymphocyte levels in the lung are not only highly statistically significant but also strong in relative terms.

10.1128/mSystems.00741-20.6 FIG S5 Correlations between baseline levels of cytotoxic lymphocytes and CCL4 and CCL5 in human lung tissue. The correlations between baseline levels of the chemokines CCL4, CCL5, and CD8^+^ T cells, resting and activated NK cells in human lung tissue are shown. Data are from the GTEx data set (*n* = 578). *R* and *P* values are from Spearman correlations. See also Fig. 2g. Download FIG S5, PDF file, 0.8 MB. Copyright © 2020 Duijf. 2020 Duijf This content is distributed under the terms of the Creative Commons Attribution 4.0 International license.

10.1128/mSystems.00741-20.9 TABLE S3 Meta-analysis of ACE2 mRNA and protein levels in 17 human tissues. Download Table S3, DOCX file, 0.06 MB. Copyright © 2020 Duijf. 2020 Duijf This content is distributed under the terms of the Creative Commons Attribution 4.0 International license.

Further, how well ACE2 mRNA and protein levels correlate was tested. Using the mRNA and protein levels in 52 cell lines, ACE2 mRNA levels were found to strongly correlate with ACE2 protein levels in human cells (Pearson *R *= 0.8155, *P* = 1.8 × 10^−13^; Fig. 2c). In fact, ACE2 ranks in the top 1.8 percentile of over 12,000 genes with the strongest mRNA-protein level correlations (*P* < 2.2 × 10^−16^; Fig. 2d). Using two ACE2-specific antibodies, immunochemistry on 40 human tissues also shows a strong ACE2 mRNA-protein correlation (*P* = 0.0011, Kruskal-Wallis test; Fig. 2e), and this is additionally validated by a meta-analysis conducted using nine published studies (Pearson *R *= 0.7130, *P* = 0.0013; Fig. 2f and Table S3). Therefore, I conclude that ACE2 mRNA and protein levels very strongly correlate, both in human cells and in human tissues.

Above, I found that baseline ACE2 levels in the lung negatively correlate with CD8^+^ T cells and resting and activated NK cells in multivariate analyses and in an independent data set (Fig. 1a to f). Several cytokines, including C-C motif chemokine ligand 2 (CCL2) to CCL5, C-X-C motif chemokine ligand 9 (CXCL9), CXCL10, CXCL16, and interleukin 18 (IL-18), are known to chemotactically attract CD8^+^ T cells and NK cells (32–37). Consistently, I find that the baseline levels of these chemokines in human lung tissue typically significantly correlate with the baseline levels of CD8^+^ T cells and resting and activated NK cells (Fig. 2g and Fig. S5). Additionally, as expected given the results above, significant negative correlations were found between the levels of ACE2 and the levels of six of these eight cytokines in the lung (Fig. 2h). These findings lend further support to my previous observations, suggesting that high levels of said cytokines in the lung establish a favorable milieu for cytotoxic lymphocytes, which correlates with low ACE2 levels.

Next, the direct consequences of SARS-CoV-2 infection were assessed. *In vitro* SARS-CoV-2 infection of human lung cells invariably leads to upregulation of all eight above-mentioned CD8^+^ T cell- and NK cell-attracting cytokines, with six of these increases showing statistical significance (Fig. 2i). Similarly, compared to control lung tissues, all eight cytokines are upregulated in lung tissues from COVID-19 patients, with five showing statistical significance (Fig. 2j). Moreover, the levels of CD8^+^ T cells and NK cells are higher in bronchoalveolar lavage fluids from mildly affected COVID-19 patients than from severe cases, with CD8^+^ T cells and a subset of NK cells, inflammatory NK cells, showing a statistically significant higher level (Fig. 2k). These findings are corroborated in a different cohort of patients, additionally showing a highly significant increase in a tissue-resident signature score for CD8^+^ T cells (Fig. 2k). Thus, together, these observations suggest that SARS-CoV-2 infection of lung cells stimulates CD8^+^ T cell- and NK cell-attracting cytokines and that these cytotoxic lymphocytes are important for preventing severe symptoms of COVID-19.

**FIG 3:**
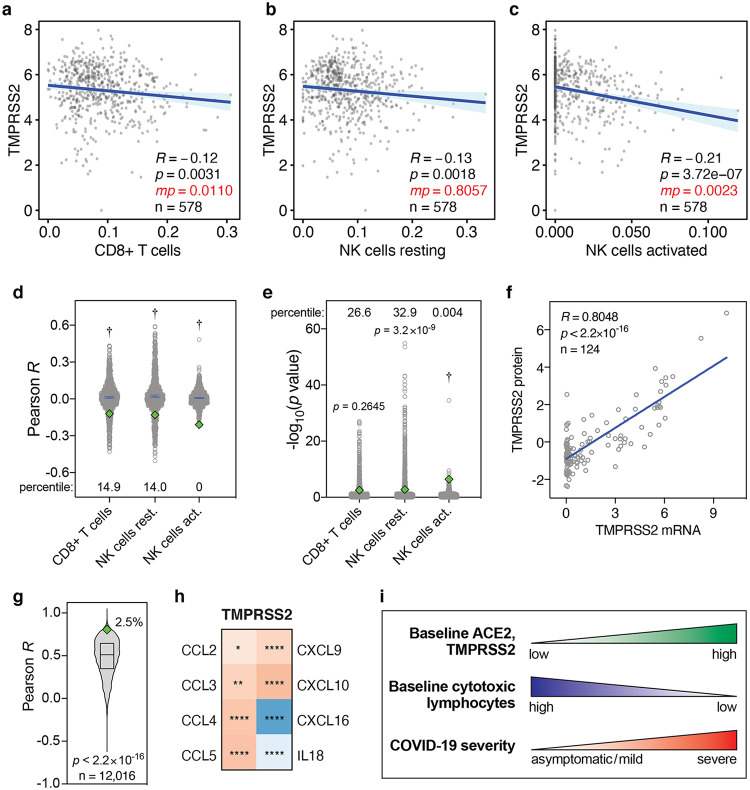
Levels of TMPRSS2 mRNA, TMPRSS2 protein, and cytokines in lung cells and tissues. (a to c) Pearson correlations between baseline levels of indicated lymphocytes and TMPRSS2 in human lung tissue, as in Fig. 1a to c. Data are from the GTEx data set (*n* = 578). (d and e) Pearson *R* and −log_10_ *P* values of correlations between 1,000 randomly sampled genes and the levels of indicated lymphocytes in lung tissues, as in Fig. 2a and b. TMPRSS2 Pearson *R* and *P* values are shown as green diamonds. (f) Correlations between TMPRSS2 mRNA and protein levels in 124 cell lines. *R* and *P* values are from Pearson correlations. (g) Pearson correlation *R* values between mRNA and protein levels of 12,016 genes are compared to the TMPRSS2 *R* coefficient (green diamond). The line and box represent the median and interquartile ranges, respectively. The TMPRSS2 *R* percentile is also shown. The *P* value is from one-sample *t* test. (h) Heatmap showing Spearman correlations between the levels of TMPRSS2 and cytokines in human lung tissues from the GTEx data set (*n* = 578), as in Fig. 2h. (i) Individuals with high baseline levels of ACE2 and TMPRSS2 show low baseline tissue-resident levels of cytotoxic lymphocytes in the lung. I propose that this may jointly predispose these individuals to development of severe COVID-19. *P* value symbols: *, *P* < 0.05; **, *P* < 0.01; ****, *P* < 0.0001; †, *P* < 2.2 × 10^−16^.

Finally, whether the levels of the SARS-CoV-2 host cell protease TMPRSS2 shows similar correlations with the levels of CD8^+^ T cells and NK cells in the lung was tested. In univariate analyses, baseline TMPRSS2 levels in the lung show significant negative correlations with these lymphocyte levels, although in multivariate analyses, these correlations are statistically significant only for CD8^+^ T cells and activated NK cells (Fig. 3a to c). The corresponding *R* and *P* values are typically also significantly lower than expected by chance (Fig. 3d and e). Furthermore, TMPRSS2 mRNA and protein levels strongly correlate (*R *= 0.8048, *P* < 2.2 × 10^−16^, Fig. 3f), and TMPRSS2 is in the top 2.5 percentile of genes that show the strongest mRNA-protein correlation (*P* < 2.2 × 10^−16^, Fig. 3g). Additionally, TMPRSS2 expression tends to correlate negatively with CD8^+^ T cell- and NK cell-attracting cytokines (Fig. 3h). Therefore, albeit typically to a lesser extent, baseline TMPRSS2 expression levels in the lung negatively correlate with the levels of CD8^+^ T cells and NK cells in a manner similar to ACE2.

Taken together, these observations suggest that a subgroup of individuals may be exceedingly susceptible to developing severe COVID-19 due to concomitant high preexisting ACE2 and TMPRSS2 expression and low baseline levels of CD8^+^ T cells and NK cells in the lung (Fig. 3i).

## DISCUSSION

I investigated the baseline expression levels of the SARS-CoV-2 host cell entry receptor ACE2 and the host cell entry protease TMPRSS2 and the baseline levels of all seven types of antiviral leukocytes in 1,927 human lung tissue samples. Although SARS-CoV-2 cellular tropism is broad (16–18), I focused on lung tissue. In addition to epithelial cells elsewhere in the respiratory tract, alveolar epithelial cells are thought to be a primary SARS-CoV-2 entry point (16, 28). Consistently, the SARS-CoV-2 receptor ACE2 is expressed in these cells at the mRNA and protein levels (28, 38–40). Moreover, in severely affected COVID-19 patients, the lungs are among the few organs that present with the most life-threatening symptoms. “Cytokine storm”-induced acute respiratory distress syndrome (ARDS), widespread alveolar damage, pneumonia, and progressive respiratory failure have been observed (41, 42). These indications frequently require admission to intensive care units (ICUs), and mechanical ventilation and may ultimately be fatal.

Early after infection, rapid activation of the innate immune system is of paramount importance for the clearance of virus infections. Infected cells typically do so through release of proinflammatory cytokines and chemokines, in particular type I and III interferons (19, 20). Notably, however, several studies have highlighted multiple complexities related specifically to SARS-CoV2 and innate immune system activation at early stages. First, unlike SARS-CoV, SARS-CoV-2-infected lung tissue initially fails to induce a range of immune cell-recruiting molecules, including several interferons (17, 27), suggesting that leukocytes are ineffectively recruited to the infected lung shortly after infection. Second, the host cell entry receptor *ACE2* has been identified as an interferon target gene (28). Thus, even when interferons are upregulated in order to recruit immune cells, concomitant upregulation of ACE2 expression may in fact exacerbate SARS-CoV-2 infection (28).

These findings suggest that the levels of immune cells that already reside in the lung prior to infection may be more critical for dampening SARS-CoV-2 infection at early stages than they are for fighting infections of other viruses. Cytotoxic lymphocytes, including CD8^+^ T cells and NK cells, are key early responders to virus infections, and these are the cells whose baseline levels were identified here as significantly reduced in lung tissue with elevated ACE2 and TMPRSS2 expression. That these immune cells are important in preventing severe COVID-19 is supported by the fact that their levels are significantly higher in bronchoalveolar lavage fluids from patients with mild cases than from patients with severe cases. Therefore, our results suggest that individuals with increased baseline susceptibility to SARS-CoV-2 infection in the lungs may also be less well equipped from the outset to mount a rapid antiviral cellular immune response (Fig. 3i).

Several observations indicate that these cytotoxic lymphocytes are critically important for effective control of SARS-CoV-2 infection. Recent studies showed that CD8^+^ T cells in peripheral blood are considerably reduced and functionally exhausted in COVID-19 patients, in particular in elderly patients and in severely affected patients that require ICU admission (43–45). Reduced CD8^+^ T cell counts also predict poor COVID-19 patient survival (43). Additionally, CD8^+^ T cell- and NK cell-attracting cytokines are upregulated in SARS-CoV-2-infected human lung cells and in lung tissues from COVID-19 patients, and the levels of CD8^+^ T cells and NK cells are higher in bronchoalveolar lavage fluids from mildly affected COVID-19 patients than in patients with severe disease (46, 47).

The five phenotypic parameters, sex, age, BMI, race, and smoking history, did not statistically significantly contribute to variation in ACE2 expression in human lung tissue, either in univariate or in multivariate analyses. This is consistent with some studies but inconsistent with others (42, 48–50). These paradoxical observations may be partially explained by differing gender, age, and race distributions within each study cohort.

Further research will be required to elucidate the precise mechanisms of SARS-CoV-2-induced activation of the innate immune system early after infection. However, the link identified here between high baseline ACE2 and TMPRSS2 expression and reduced cytotoxic lymphocyte levels in human lung tissue prior SARS-CoV-2 infection is striking. It suggests that increased susceptibility to SARS-CoV-2 infection in the lungs may be accompanied by a poorer ability to mount a rapid innate immune response at early stages. This may predict long-term outcome of individuals infected with SARS-CoV-2, given that the levels of CD8^+^ T cells and NK cells are significantly higher in bronchoalveolar lavage fluids from mild cases compared to severe patients (46, 47). Finally, it may contribute to the substantial variation in COVID-19 clinical presentation, ranging from asymptomatic to severe respiratory and other symptoms.

## MATERIALS AND METHODS

### Discovery data set and processing.

Gene expression data and corresponding phenotype data from human lung tissues (*n* = 578) were obtained from the Genotype-Tissue Expression (GTEx) Portal (https://gtexportal.org), managed by the National Institutes of Health (NIH). Gene expression data were publicly available. Access to phenotype data required authorization. The GTEx protocol was previously described (29, 30). Briefly, total RNA was extracted from tissue. Following mRNA isolation, cDNA synthesis, and library preparation, samples were subjected to HiSeq2000 or HiSeq2500 Illumina TrueSeq RNA sequencing. Gene expression levels were obtained using RNA-SeQC v1.1.9 (51) and expressed in transcripts per million (TPM). Reported gene-level expression levels were log_2_ transformed, unless indicated otherwise.

### Validation data set and processing.

For validation purposes, the Laval University, University of British-Columbia, Groningen University (LUG) lung tissue data set (*n* = 1,349) was used. This data set was accessed via Gene Expression Omnibus (GEO; https://www.ncbi.nlm.nih.gov/geo), accession number GSE23546, and was previously described (52). Briefly, total RNA from human lung tissue samples was isolated, quantified, quality checked, and used to generate cDNA, which was amplified and hybridized to Affymetrix gene expression arrays. Arrays were scanned, and probe-level gene expression values were normalized using robust multichip average (RMA). These normalized values were obtained from GEO. To collapse probe-level expression data to single expression levels per gene, for each gene the probe with the highest median absolute deviation (MAD) was used. The MAD for each probe *p* was calculated using equation 1

(1) $$\text{MAD}\left(p\right)=M\left(\left|p_{i}-M\left(p\right)\right|\right)$$

where *M* is the median, *p_i_* denotes probe *p*'s expression level in sample *i*, and *M* (*p*) represents the median signal of probe *p*.

### *In silico* cytometry.

The levels of seven types of leukocytes involved in antiviral cellular immune response, specifically CD8^+^ T cells, resting NK cells, activated NK cells, M1 macrophages, CD4^+^ T cells, dendritic cells, and neutrophils, were estimated in the discovery and validation lung tissue samples using a previously described approach (31). Specifically, the following workflow was used. First, only non-log-transformed expression values were used. Thus, where required, expression values for all samples in the discovery and validation data sets were reverse log_2_ transformed using equation 2

(2) $$c=2^{c_{l}}-1$$

where *c* denotes the calculated non-log_2_-transformed expression counts and *c_l_* denotes the previously reported log_2_-transformed expression counts. Next, to compensate for potential technical differences between signatures and bulk sample gene expression values due to interplatform variation, bulk mode batch correction was applied. To ensure robustness, deconvolution was statistically analyzed using 100 permutations. Pearson correlation coefficients *R*, root mean squared errors (RMSA), and *P* values are reported on a per-sample level in Fig. S1a to c and Table S1 in the supplemental material.

### Univariate statistical analyses.

Log_2_-transformed expression levels of ACE2 and TMPRSS2 in lung tissue samples were compared to the estimated levels of seven leukocyte types or states. Pearson correlation analyses were performed to determine Pearson correlation coefficients *R* and *P* values. *P* values were adjusted at a false discovery rate of 0.05 to yield *q* values, as previously described (53). Straight lines represent the minimized sum of squares of deviations of the data points with 95% confidence intervals shown. Continuous phenotypic covariates were analyzed in the same way and, additionally, as discrete ordinal categories after binning. Discrete and binned phenotype data were statistically evaluated using Mann-Whitney *U* tests. All analyses were performed in the *R* computing environment (*R* Project for Statistical Computing, Vienna, Austria).

### Multivariate regression analyses.

Multivariate analyses were performed using standard ordinary least-squares regression, summarized in equation 3.

(3) $$Y={\text{β}}_{0}+\overset{n}{\underset{k=1}{\sum }}({\text{β}}_{k}X_{k})+\text{ε}$$

where *β*_0_ denotes the intercept, while *β_k_* represents the slope of each variable *X_k_* in a model with *n* variables and ε denotes the random error component. These analyses were performed using *R*.

### mRNA and protein levels in human cells.

Available ACE2 and TMPRSS2 mRNA and corresponding protein levels in 52 and 124 human cell lines, respectively, were obtained from references 54 and 55. The correlations between mRNA and protein levels were analyzed by linear regression analysis using Pearson correlations. Pearson coefficients *R* for mRNA-protein level correlations were also determined for 12,015 other genes. To test whether the ACE2 and TMPRSS2 coefficients and *P* values were statistically significantly lower than for other genes, one-sample *t* tests were used.

### mRNA and protein levels in human tissues.

Two types of analyses were performed to compare mRNA and protein levels in human tissues. First, mRNA expression levels from 40 human tissues were obtained from the Human Protein Atlas (https://www.proteinatlas.org). These levels represented the consensus normalized mRNA expression levels from three sources, specifically, Human Protein Atlas in-house RNAseq data, RNAseq data from the Genotype-Tissue Expression (GTEx) project and CAGE data from FANTOM5 project. Corresponding ordinal human tissue protein expression levels (“not detected,” “low,” and “'high”) were also obtained from the Human Protein Atlas. These levels were based on immunohistochemical staining of the tissues using DAB (3,3′-diaminobenzidine)-labeled antibodies (HPA000288 and CAB026174), followed by knowledge-based annotation, as described on the website. A Kruskal-Wallis test was performed to assess whether mRNA and protein levels significantly correlated.

Second, a meta-analysis was performed. For this, mRNA and protein expression levels were obtained from nine different sources. Tissue mRNA levels were obtained from six sources, as determined by Northern blotting (56), quantitative reverse transcription-PCR (RT-PCR) (57), microarray hybridization (58), RNA sequencing (59), and cap analysis of gene expression (60). Tissue protein levels were obtained from three sources, as determined by mass spectrometry (61) and immunohistochemistry (39, 62). For each source and tissue, mRNA and protein expression levels were scored as “not detected,” “low,” “intermediate,” or “high” and these received scores of 0 to 3, respectively. For each tissue, the final mRNA and protein scores were calculated by averaging the scores from the respective six and three sources (Table S3). Strength and significance level of the correlation between the final scores were determined by linear regression analysis using Pearson correlation.

### Cytokine levels in SARS-CoV-2-infected lung cells.

SARS-CoV-2-induced fold increases in the expression levels of eight cytotoxic lymphocyte-attracting cytokines, CCL2-5, CXCL9, CXCL10, CXCL16, and IL-18, were determined from reference 27. These increases represent the fold increase in expression in Calu-3 lung cells, 24 h after infection with SARS-CoV-2 at a multiplicity of infection of 2, compared to uninfected Calu-3 cells.

### Cytokine levels in control and COVID-19 lung tissues.

Baseline levels of the above eight cytokines in lung tissues were obtained from the GTEx project, as described above, and compared to the baseline levels of ACE2, TMPRSS2, CD8^+^ T cells, resting and activated NK cells in these tissues, estimated as described above. Their levels were compared using Spearman’s rank correlations (*R* and *P* values). For comparison of cytokine levels in postmortem COVID-19 lung tissues (*n* = 2) to those in healthy, uninfected lung tissues (*n* = 2), fold increases were determined following RNAseq analyses and previously reported (27).

### Lymphocyte levels in COVID-19 bronchoalveolar lavage fluid samples.

The levels of CD8^+^ T cells, NK cells, and inflammatory NK cells in bronchoalveolar lavages from mild (*n* = 2) and severe (*n* = 22) COVID-19 patients were reported elsewhere and determined using single-cell RNAseq (46). Statistical significance levels were assessed using Mann-Whitney *U* tests. The levels of T cells and NK cells, as well as a CD8^+^ T cell tissue-resident signature score, in bronchoalveolar lavage fluid samples from moderate (*n* = 3) and severe/critical (*n* = 5) COVID-19 patients were reported in another study (47).

### Data availability.

The data sets used for the analyses described in this study were obtained from dbGaP at https://www.ncbi.nlm.nih.gov/gap through dbGaP accession number phs000424.v8.p2.

10.1128/mSystems.00741-20.1 TEXT S1 Supplemental references. Download Text S1, DOCX file, 0.01 MB. Copyright © 2020 Duijf. 2020 Duijf This content is distributed under the terms of the Creative Commons Attribution 4.0 International license.
