# Supplementary figures and images for: Low Baseline Pulmonary Levels of Cytotoxic Lymphocytes as a Predisposing Risk Factor for Severe COVID-19
Source: mSystems. 2020 Sep 1;5(5):e00741-20. doi: 10.1128/mSystems.00741-20 (PMC7470988; doi:10.1128/mSystems.00741-20)

Figure S1

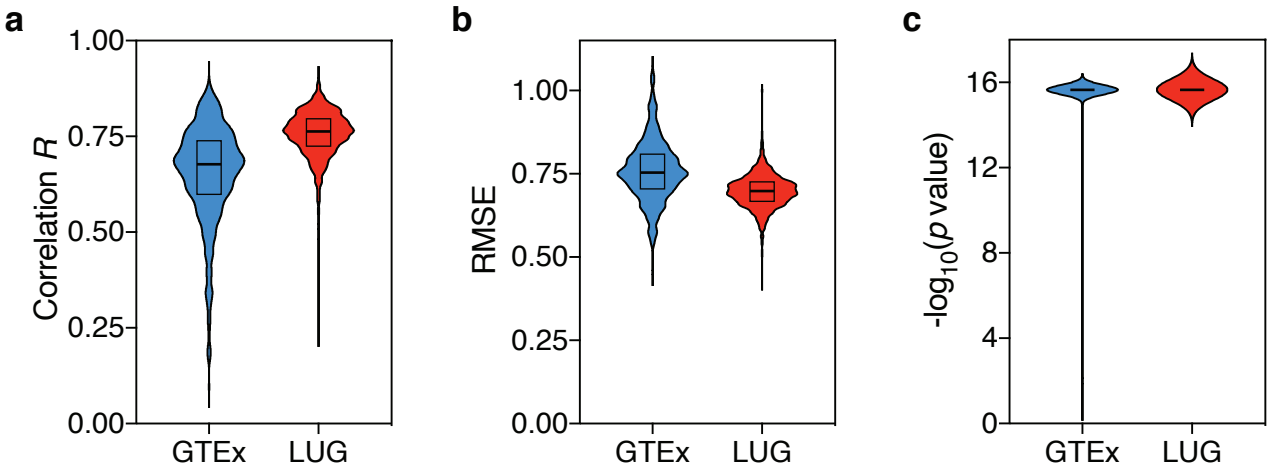

Supplement: FIG S1 [file mSystems.00741-20-sf001.pdf]

**Figure S2**

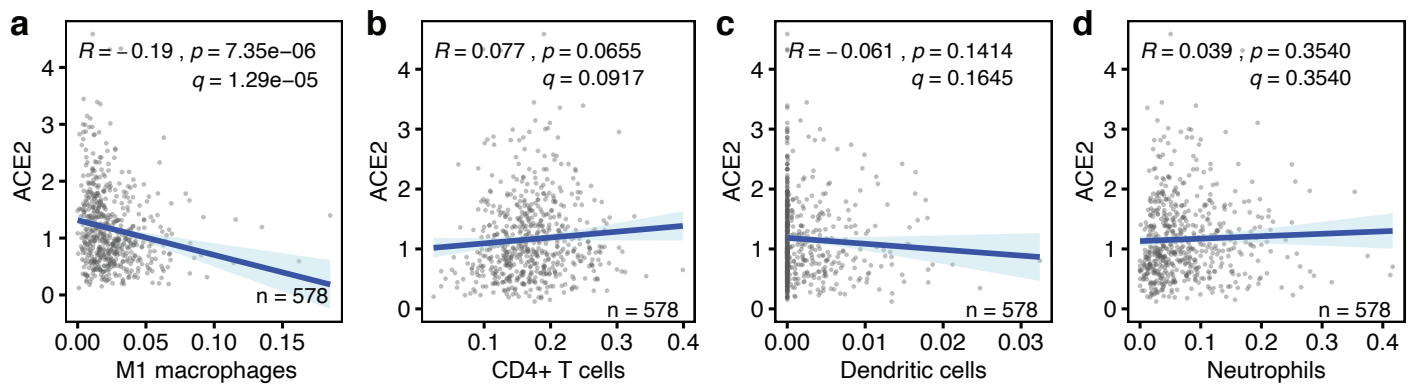

Supplement: FIG S2 [file mSystems.00741-20-sf002.pdf]

Figure S3

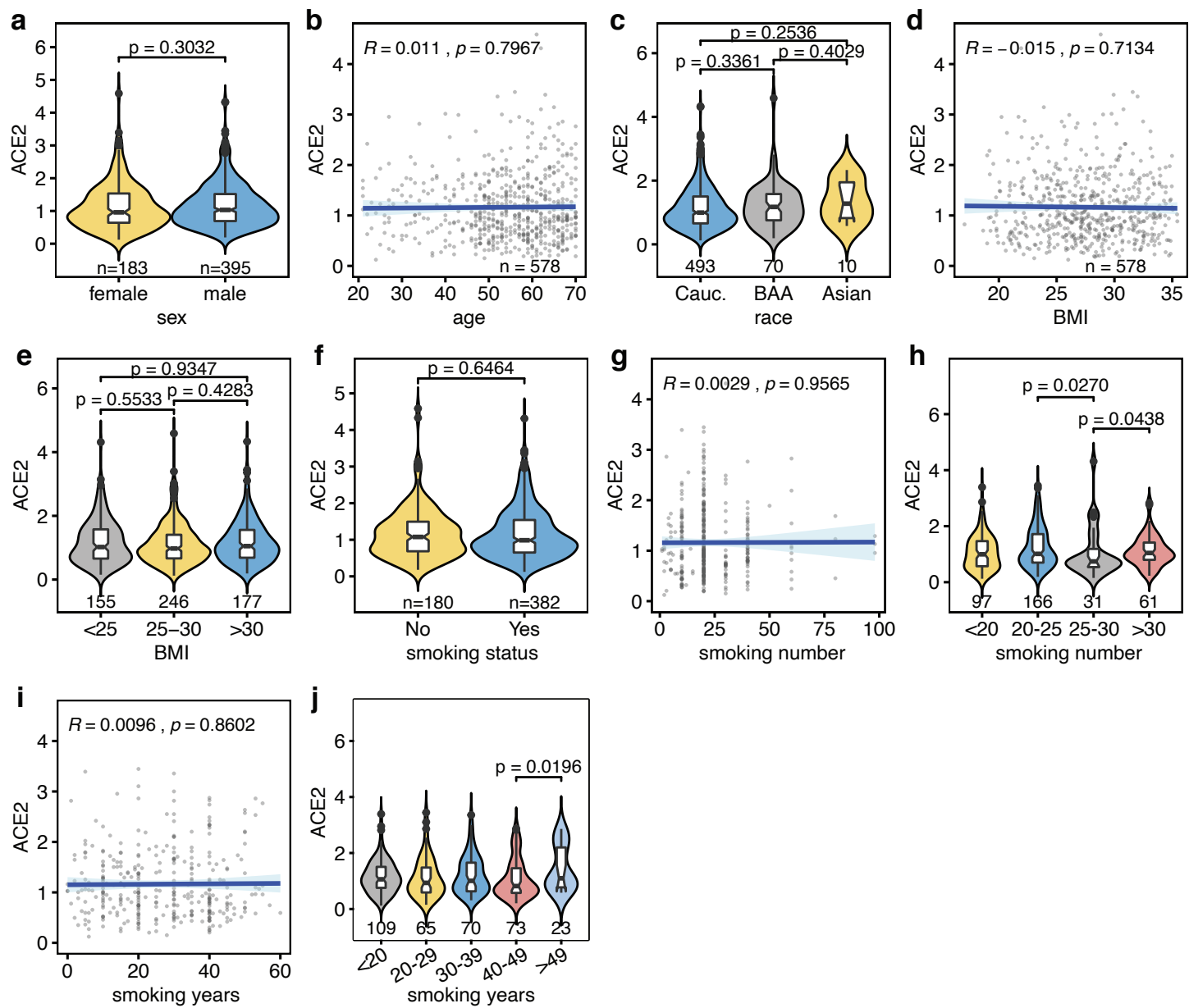

Supplement: FIG S3 [file mSystems.00741-20-sf003.pdf]

**Figure S4**

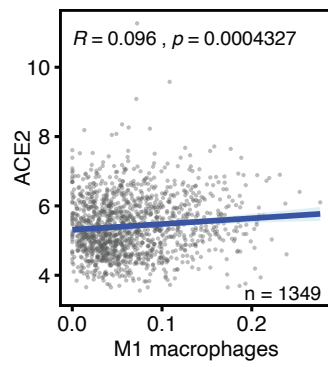

Supplement: FIG S4 [file mSystems.00741-20-sf004.pdf]

Figure S5

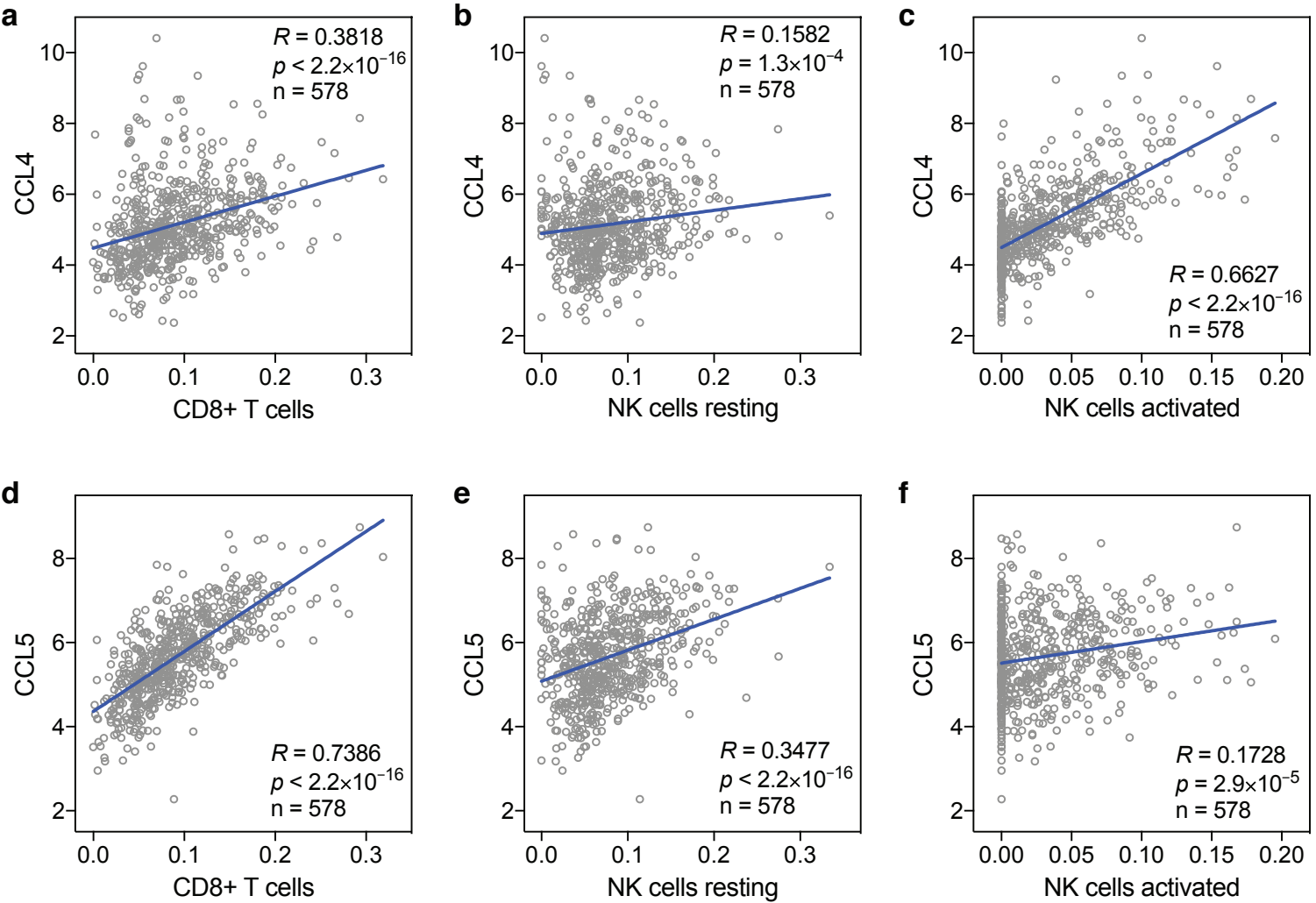

Supplement: FIG S5 [file mSystems.00741-20-sf005.pdf]
