# Supplementary material for: Low Baseline Pulmonary Levels of Cytotoxic Lymphocytes as a Predisposing Risk Factor for Severe COVID-19
Source: mSystems. 2020 Sep 1;5(5):e00741-20. doi: 10.1128/mSystems.00741-20 (PMC7470988; doi:10.1128/mSystems.00741-20)
Supplement: TABLE S2 [file mSystems.00741-20-st002.docx]

| **Table S2. Phenotypic characteristics of GTEx lung tissue donors** | | |
| --- | --- | --- |
| **Feature** | **Number of individuals (%)** | **Median (range)** |
| Sex  Female  Male | 183 (31.7)  395 (68.3) |  |
| Age  <40  40-49  50-59  60-69  >69 | 73 (12.6)  93 (16.1)  200 (34.6)  190 (32.9)  22 (3.8) | 56 (21 - 70) |
| Body mass index  <25  25-30  >30 | 155 (26.8)  246 (42.6)  177 (30.6) | 27.7 (17.0 - 35.4) |
| Race  Caucasian  Black/African American  Asian  American Indian/Alaska Native | 493 (85.9)  70 (12.2)  10 (1.7)  1 (0.2) |  |
| Smoking status  Non-smoker  Smoker | 180 (32.0)  382 (68.0) |  |
