# Supplementary material for: Low Baseline Pulmonary Levels of Cytotoxic Lymphocytes as a Predisposing Risk Factor for Severe COVID-19
Source: mSystems. 2020 Sep 1;5(5):e00741-20. doi: 10.1128/mSystems.00741-20 (PMC7470988; doi:10.1128/mSystems.00741-20)
Supplement: TABLE S3 [file mSystems.00741-20-st003.docx]

| **Table S3. Meta-analysis of ACE2 mRNA and protein levels in 17 human tissues**  *Source data of Fig. 2f.* | | | | | | | | | | | | | | | | | | | |
| --- | --- | --- | --- | --- | --- | --- | --- | --- | --- | --- | --- | --- | --- | --- | --- | --- | --- | --- | --- |
| Source data type | Source data method | Adipose tissue | Airway epithelium | Brain | Cardiac muscle | Colorectal tissue | Endometrium | Gall bladder | Kidney | Liver | Lung | Oral mucosa/esophagus | Skin | Small intestine | Smooth muscle | Spleen | Stomach | Testis | Ref. |
| mRNA | NB | 0 | N | N | 3 | 2 | 0 | N | 3 | 0 | 0 | N | 0 | 2 | 0 | N | N | 3 | [1] |
| mRNA | qRT-PCR | 1 | 1 | 0 | 2 | 2 | 1 | 2 | 2 | 1 | 1 | 1 | 0 | 3 | 1 | 0 | 1 | 3 | [2] |
| mRNA | MA | 2 | 2 | 2 | 2 | 2 | 1 | N | 3 | 2 | 2 | N | 1 | 3 | 2 | N | N | 3 | [3] |
| mRNA | RNAseq | 2 | N | 2 | 2 | 1 | 1 | 3 | 3 | 0 | 0 | 0 | 1 | 3 | 2 | N | 0 | 3 | [4] |
| mRNA | RNAseq | N | N | N | N | N | N | N | N | N | N | 1 | N | N | N | N | N | N | [5] |
| mRNA | CAGE | 0 | N | 0 | 1 | 2 | 0 | 1 | 1 | 0 | 0 | 0 | N | 3 | 0 | 0 | N | 2 | [6] |
| Protein | MS | N | N | 0 | 1 | 0 | N | 1 | 3 | 1 | 0 | 0 | N | N | N | N | N | 3 | [7] |
| Protein | IHC | 3 | 1 | 1 | N | 1 | N | N | 3 | 1 | 3 | 2 | 3 | 3 | 3 | 3 | 0 | N | [8] |
| Protein | IHC | 0 | 0 | 0 | 1 | 2 | 0 | 3 | 3 | 0 | 0 | 0 | 0 | 3 | 0 | 0 | 0 | 3 | [9] |
| **Scores (averages)** | | | | | | | | | | | | | | | | | | | |
| **mRNA score** | | 1.0 | 1.5 | 1.0 | 2.0 | 1.8 | 0.6 | 2.0 | 2.4 | 0.6 | 0.6 | 0.5 | 0.5 | 2.8 | 1.0 | 0.0 | 0.5 | 2.8 |  |
| **Protein score** | | 1.5 | 0.5 | 0.3 | 1.0 | 1.0 | 0.0 | 2.0 | 3.0 | 0.7 | 1.0 | 0.7 | 1.5 | 3.0 | 1.5 | 1.5 | 0.0 | 3.0 |  |
| *Abbreviations:* CAGE, cap analysis of gene expression; IHC, immunohistochemistry; MA, microarray; MS, mass spectrometry; N, not determined; NB Northern blot. | | | | | | | | | | | | | | | | | | | |
