# Supplementary material for: Low Baseline Pulmonary Levels of Cytotoxic Lymphocytes as a Predisposing Risk Factor for Severe COVID-19
Source: mSystems. 2020 Sep 1;5(5):e00741-20. doi: 10.1128/mSystems.00741-20 (PMC7470988; doi:10.1128/mSystems.00741-20)
Supplement: TEXT S1 [file mSystems.00741-20-s0001.docx]

**Supplementary References**

1. Tipnis, S.R., et al., A human homolog of angiotensin-converting enzyme. Cloning and functional expression as a captopril-insensitive carboxypeptidase. J Biol Chem, 2000. 275(43): p. 33238-43.

2. Harmer, D., et al., Quantitative mRNA expression profiling of ACE 2, a novel homologue of angiotensin converting enzyme. FEBS Lett, 2002. 532(1-2): p. 107-10.

3. Wu, C., et al., BioGPS: building your own mash-up of gene annotations and expression profiles. Nucleic Acids Res, 2016. 44(D1): p. D313-6.

4. Uhlen, M., et al., A pathology atlas of the human cancer transcriptome. Science, 2017. 357(6352).

5. GTEx Consortium, Genetic effects on gene expression across human tissues. Nature, 2017. 550(7675): p. 204-213.

6. FANTOM Consortium, RIKEN PMI, CLST (DGT), A promoter-level mammalian expression atlas. Nature, 2014. 507(7493): p. 462-70.

7. Kim, M.S., et al., A draft map of the human proteome. Nature, 2014. 509(7502): p. 575-81.

8. Hamming, I., et al., Tissue distribution of ACE2 protein, the functional receptor for SARS coronavirus. A first step in understanding SARS pathogenesis. J Pathol, 2004. 203(2): p. 631-7.

9. Thul, P.J., et al., A subcellular map of the human proteome. Science, 2017. 356(6340).
